# Luminal acetylation of microtubules is not essential for *Plasmodium berghei* and *Toxoplasma gondii* survival

**DOI:** 10.15698/mic2025.12.863

**Published:** 2025-12-17

**Authors:** Thrishla Kumar, Katharina Röver, Johannes F. Stortz, Annika M. Binder, Benjamin Spreng, Madlen Konert, Markus Meissner, Friedrich Frischknecht, Elena Jimenez-Ruiz

**Affiliations:** 1 Experimental Parasitology, Veterinary Medicine Faculty, Ludwig-Maximilians-Universität Munich, Germany; 2 Parasitology, Center for Infectious Diseases, Medical Faculty, Heidelberg University, Im Neuenheimer Feld 344, 69120 Heidelberg, Germany; 3 Centre for Parasitology, University of Glasgow, United Kingdom; 4 German Center for Infectious Diseases, partner site Heidelberg

**Keywords:** microtubule acetylation, K40 acetylation, tubulin modifications, apicomplexan parasites, parasite cytoskeleton

## Abstract

Post-translational modifications of microtubules regulate their stability and dynamics. Acetylation of α tubulin at lysine 40 (K40) by α -acetyltransferase ( α TAT) occurs on the luminal side of microtubules, stabilizes their structure, and plays essential roles in various cellular processes across eukaryotes. Apicomplexan parasites include the malaria-causing *Plasmodium* species and *Toxoplasma gondii*, both of which possess unusually stable subpellicular microtubules, a set of cytoskeletal filaments underlying the parasite’s inner membrane complex. Interestingly, while *Toxoplasma gondii* and human-infecting *Plasmodium* species retain both K40 and α TAT, rodent-infecting *Plasmodium* species have lost α TAT, and K40 has been replaced by glutamine (Q40), a residue that can mimic acetylated lysine. Here, we investigate the role of microtubule acetylation in apicomplexan parasites by generating and characterizing genetic mutants in *Plasmodium berghei* and *Toxoplasma gondii*. In *Plasmodium berghei*, introduction of a Q40K mutation in α 1 tubulin did not affect parasite development or infectivity, suggesting that the absence of K40 acetylation is not detrimental. In *Toxoplasma gondii*, we confirmed that α TAT is responsible for microtubule acetylation but, contrary to previous reports, its deletion had no impact on parasite growth *in vitro*. Together, these results indicate that luminal K40 acetylation is not essential for microtubule function in either species, pointing to functional redundancy and highlighting the plasticity of cytoskeletal regulation in apicomplexan parasites.

## INTRODUCTION

Apicomplexan parasites are the causative agents of infectious diseases such as malaria (*Plasmodium* spp.), toxoplasmosis (*Toxoplasma gondii*), and diarrhoea (*Cryptosporidium* spp.), which can severely affect both human health and livestock. These single-celled eukaryotes exhibit highly divergent biology, including unique cytoskeletal features like specialized microtubule arrays 1. In these parasites, microtubules vary in stability depending on their function: spindle and hemi-spindle microtubules are dynamic, while the subpellicular microtubules (SPMTs) in extracellular stages are highly stable 2. Extracellular stages, such as *T. gondii* tachyzoites and *Plasmodium* sporozoites, are motile cells that cross tissue barriers or invade host cells using an actin-myosin machinery 3–5. In *T. gondii*, the tachyzoite stage contains 22 SPMTs that encase secretory organelles and vesicles. These microtubules are nucleated at the apical polar ring and underlie the inner membrane complex, a unique double membrane structure beneath the parasite plasma membrane 6–8. In *Plasmodium*, the ookinete and sporozoite stages rely on specialized microtubules for their structure and motility. Ookinetes possess over 50 SPMTs, while sporozoites have at least 11 straight SPMTs that stabilize their slender shape and facilitate migration and invasion 9. Unlike tachyzoites and ookinetes, which move short distances *in vivo*, sporozoites travel hundreds of microns through the skin to locate and invade blood vessels 10–12.

**Figure 1:**
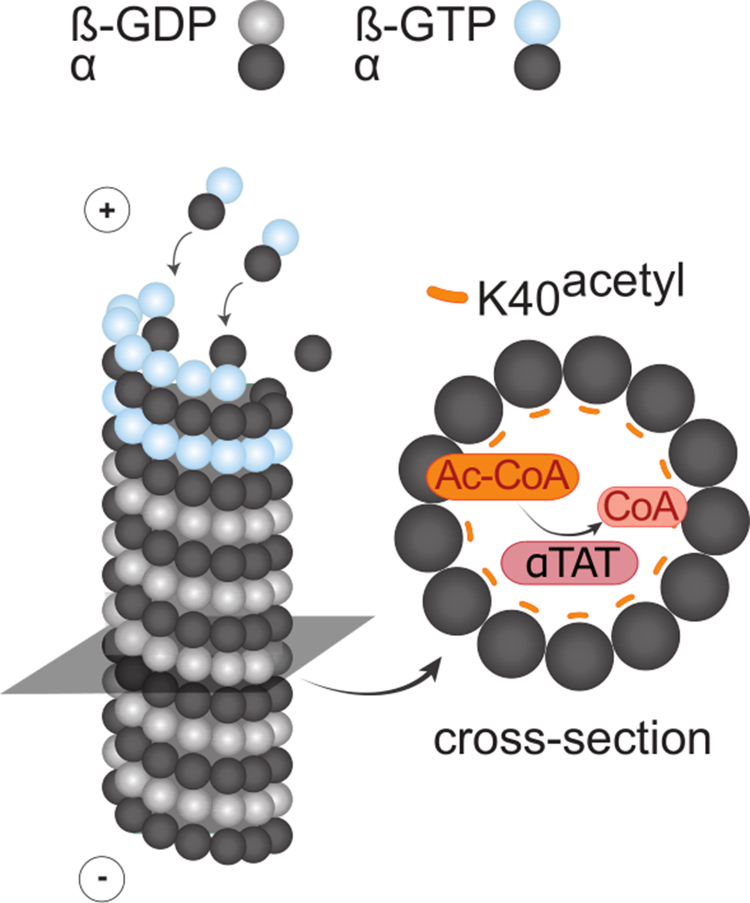
Schematic representation of microtubule acetylation. Acetylation of lysine 40 (K40) occurs on α tubulin within the microtubule lumen and is catalysed by the acetyltransferase α TAT using acetyl-CoA (Ac-CoA) as a donor. β tubulin monomers can be bound to guanosine triphosphate ( β -GTP) or guanosine diphosphate ( β -GDP). α : α tubulin monomer.

Several studies have demonstrated that apicomplexan SPMTs are remarkably stable, as evidenced by detergent extraction experiments 13, 14 and deletion of various microtubule-associated proteins 15. Notably, deletion of *spm1* (*subpellicular microtubule protein 1*) leads to a complete loss of detergent-stable SPMTs, yet results in only a moderate fitness defect in *T. gondii* 16, suggesting that detergent resistance does not necessarily reflect physiological stability *in vivo*. While luminal proteins may contribute to this stability, their deletion had minimal impact on the parasite viability, suggesting additional factors contribute to microtubule stability 14, 17. One such factor is alpha-tubulin acetyltransferase ( α TAT), an enzyme localized in the lumen of microtubules where it acetylates lysine 40 (K40) of α 1 tubulin (Figure 1). First identified in *Caenorhabditis elegans*, α TAT plays critical roles in cilia assembly and mechanosensation in other eukaryotes 18. In *T. gondii*, conflicting reports exist regarding its function. While deletion of α TAT was linked to defects in cell division, mutations at K40, the residue it modifies, did not result in a phenotype, leaving its role in parasite biology unclear 19.

In *Plasmodium berghei* and other rodent-infecting *Plasmodium* species, the lack of α TAT features a glutamine (Q) residue at position 40 in both tubulin isoforms. In contrast, human-infecting *Plasmodium* species, such as *Plasmodium falciparum*, retain α TAT and feature a K40 residue in α 1 tubulin, while α 2 tubulin carries a Q40 substitution. Synteny analysis suggests that α TAT was lost specifically in rodent-infecting lineages during evolution potentially reflecting distinct evolutionary pressures (Table 1). Since glutamine can mimic acetylated lysine 20, this substitution may compensate for the absence of α TAT. 

**Table 1:** Conservation of α tubulin K40 and presence of α TAT across apicomplexan parasites. Alignment of α tubulin isoforms from *Toxoplasma gondii* and various *Plasmodium* species. Protein sequences of α 1 and α 2 tubulin isoforms were retrieved from VEuPathDB 21 and aligned using BioEdit. Only 40 amino acids surrounding position 40 are shown, with the real positions marked on the top ruler, to highlight the relevant changes around lysine 40. The lysine or glutamine residue at position 40 is highlighted in magenta. The presence or absence of the α tubulin acetyltransferase ( α TAT) gene in each species is indicated. Species are grouped according to their host type.

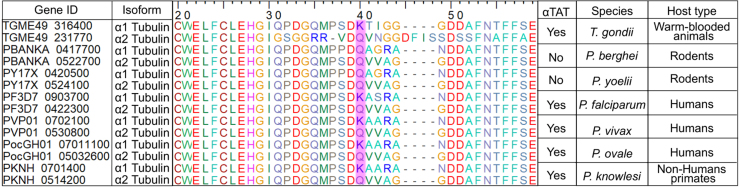

In apicomplexan parasites, the two α tubulin isoforms also differ in their expression patterns and biological roles. α 1 tubulin is expressed in asexual stages, whereas α 2 tubulin is primarily found in male gametocytes, where it contributes to axoneme formation in *P. falciparum* 9, 22. Despite both genes being essential, their functional specializations likely influence how microtubule regulation is achieved across different stages of the parasite life cycle.

We hypothesized that the Q40 residue in α 1 tubulin and the absence of α TAT in *P. berghei* reflects functional adaptations in microtubule regulation. To test this, we generated mutants in *P. berghei* that exchanged Q40 for K40 (Q40K) using CRISPR/Cas9 and classical mutagenesis. We then examined the phenotypic consequences of this substitution *in vivo*. To further investigate the role of α TAT and K40 acetylation, we also used conditional knockouts and site-directed mutagenesis in *T. gondii*. Together, these complementary approaches allowed us to test whether microtubule acetylation is required for parasite development, motility, or infectivity.

## RESULTS

### Presence of α TAT or Q40K mutation does not affect growth nor infectivity in *P. berghei*

**Figure 2:**
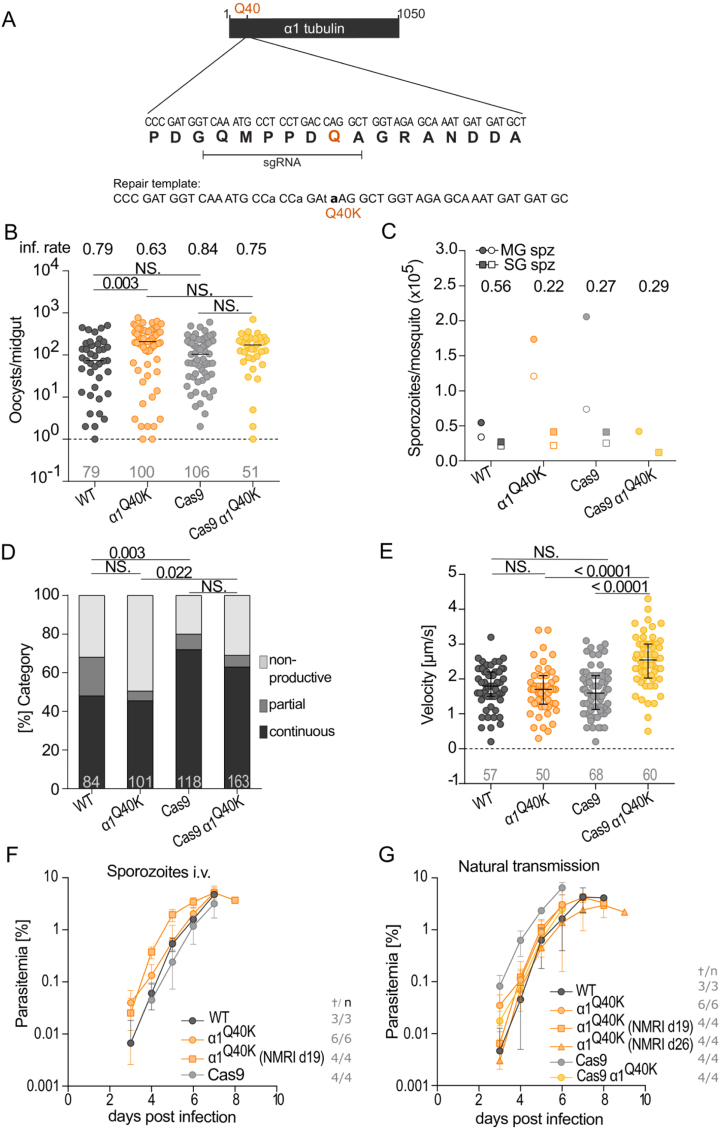
Characterization of *P. berghei* Q40K mutants. **(A)** Schematic representation of the strategy followed to create the Q40K mutant via CRISPR/Cas9. Together with the point mutations of the Q40 codon (bold and small letter), shielding mutations (indicated in small letters) were inserted via the repair template to avoid continues cleavage by Cas9. **(B)** Oocyst infection rate and colonization of *Anopheles stephensi* mosquito midguts infected by *P. berghei* WT (WT), α 1 ${}^{\mathrm{Q40K}}$ , Cas9 and Cas9 α 1 ${}^{\mathrm{Q40K}}$ parasites. Each dot represents one infected midgut, number of analysed midguts indicated below; black lines indicate median. Infection rates are indicated above. One-way ANOVA with Dunn’s test. **(C)** Midgut (MG) and salivary gland (SG) sporozoite counts. Ratios of salivary gland/midgut sporozoites are depicted at the top. Corresponding counts from one infection are indicated by the same symbol fillings. **(D)** Analysis of gliding motility patterns of salivary gland sporozoites. Motility was categorized into continuous, partial and non-productive motility. The number of sporozoites analysed for each condition is specified at the bottom of each stacked bar. Fisher’s exact test with Bonferroni Holm correction. **(E)** Velocity of continuously moving salivary gland sporozoites, number of analysed salivary gland sporozoites indicated below. Black lines indicate the median with interquartile ranges. One-way ANOVA with Dunn’s test. **(F-G)** Parasitaemia of infected mice with (F) sporozoites injected intravenously or (G) transmitted by mosquito bites. Numbers ( † /n) indicate blood-stage positive mice vs total mice. (B-G) Pooled data of two independent cage feeds (WT, n=2 ; α 1 ${}^{\mathrm{Q40K}}$ , n=2 ; Cas9, n=2 ) or one cage feed (Cas9 α 1 ${}^{\mathrm{Q40K}}$ , n=1 ). Note that WT data panels B-G were performed in parallel to our experiments and were previously published in 17, 23. Data from the different mutants were compared to its respective parental strain for statistical purposes.

To probe if the replacement of K with Q at position 40 stabilizes microtubules, the Q40K mutation was generated in α 1 tubulin of *P. berghei* via standard homologous recombination or CRISPR/Cas9-mediated mutagenesis (Figure 2 **A**; Supplementary Figure S1, S2). Structural analysis of the α 1 tubulin Q40K mutation was performed to evaluate its potential impact on protein conformation. *In silico* predictions suggest that substituting the glutamine residue at position 40 in wildtype (WT) α 1 tubulin (Supplementary Figure S3A) with lysine in the Q40K mutant (Supplementary Figure S3B) is unlikely to cause major structural deviations in the tubulin dimer. Alignment of the WT and Q40K protein structures and their predicted alignment error (PAE) scores confirmed that the mutation would not disrupt the overall dimer architecture (Supplementary Figure S3C).

Comparative analyses revealed that Q40K mutant parasites, generated either by classical insertion of the point mutation or using a Cas9-based strategy, progressed normally through mosquito development and transmission stages, showing no detectable differences compared to WT parasites (Figure 2). To assess the effect of the Q40K mutation during mosquito infection, oocyst development and midgut colonization rates were measured in *Anopheles stephensi*. The infection rates of mosquito midguts infected with WT, Q40K, and Cas9 control parasites were comparable and resulted in similar numbers of oocysts between mutants and their respective parental lines (Figure 2 **B**). Consistently, sporozoite quantification in midguts and salivary glands revealed that the Q40K mutation did not disrupt sporozoite production nor salivary gland invasion (Figure 2 **C**). While some variability in midgut sporozoite numbers was observed between conditions, this reflects the natural variability between mosquitos fed on different mice, which can differ slightly in gametocyte density and infectivity. To control for this, we included the ratio of salivary gland to midgut sporozoites, with values above 20% generally indicating productive infections. No systematic differences were seen between the mutants and their respective controls.

The motility of salivary gland sporozoites was further examined, categorizing movement into continuous, partial, or non-productive gliding patterns. Sporozoites from Q40K mutants exhibited a distribution of motility patterns indistinguishable from control line parasites (Figure 2 **D**). Additionally, the velocity of continuously gliding sporozoites was measured, with no significant differences observed between groups with the exception of the Cas9 generated Q40K, which glide slightly faster (Figure 2 **E**). Finally, the capacity of Q40K mutant sporozoites to establish infection in mice was tested by both intravenous injection and natural transmission through mosquito bites. The rates of blood-stage parasitaemia were comparable between groups, indicating the mutation does not impair the parasite’s ability to infect the host (Figure 2 **F, G**).

Overall, these data suggest that the acetylation state at position 40 of α 1 tubulin in *P. berghei* is by itself not required for conferring stability to microtubules.

### α TAT is not important for *Toxoplasma gondii*

**Figure 3:**
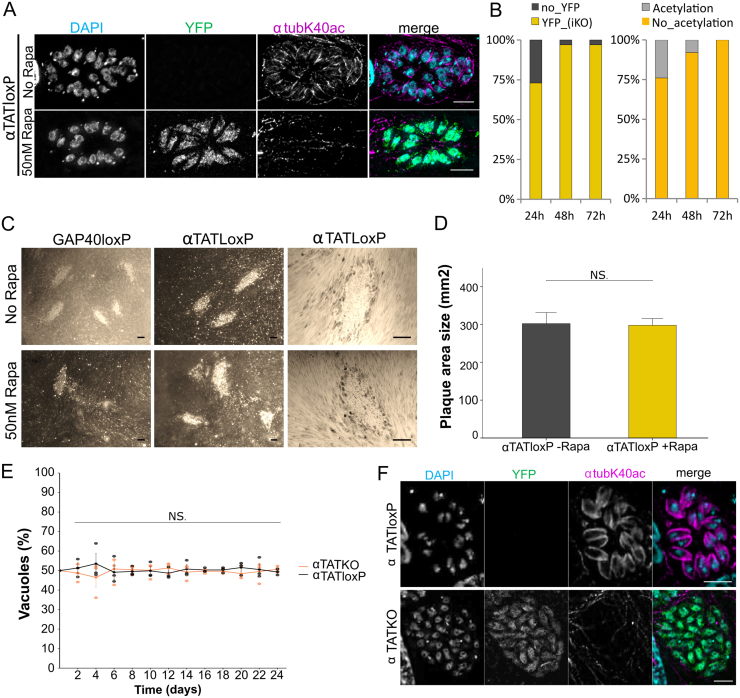
Deletion of endogenous α TAT in *T. gondii* leads to lack of acetylation in microtubules. **(A)** α *tat* gene excision with the DiCre system. IFA depicting α 1 tubulin acetylation at lysine 40 ( α tubK40ac) in RH Δ ku80-DiCre-*Pf* α *tat*-loxP ( α TATLoxP). To achieve α *tat* gene excision, parasites were incubated with 50 nM rapamycin for 4 h. Successful gene excision resulted in YFP expression (lower panel). Loss of α *tat* gene function resulted in parasites lacking α 1 tubulin acetylation. Scale bars: 5 μ m. **(B)** Measurement of rapamycin induction efficiency in the induced α TATKO parasite population. For this, a YFP signal or the absence of acetylation were exploited as a marker for successful excision of Pf α *tat* cDNA (induced knock-out: iKO). Following rapamycin induction overnight, α TATKO parasite populations were stained for YFP (left graph) or K40 α 1 tubulin acetylation (right graph) via IFA after 24 hours (h), 48 h and 72 h post induction. Subsequently, parasitic vacuoles ( n=100 ) were counted and investigated with regards to the excision markers. **(C)** Plaque assay investigating replication in α TATKO parasites. Overview pictures (left and middle panels) represent approximate plaque numbers for gap40loxP (- Rapa) and gap40KO (50 nM Rapa) as well as α TATloxP(- Rapa) and α TATKO (50 nM Rapa) parasites. Close-up pictures (right panel) show a plaque produced by α TATloxP or α TATKO parasites. Scale bars: 250 μ m. **(D)** Quantification of α TATloxP and α TATKO plaque area after 5 days post-infection ( n=30 /condition). Data shown as mean + standard deviation (SD). *t*-Student test performed. NS.: Non-significant. **(E)** α TAT Competition assay was performed over a duration of 24 days. Time progression of vacuoles ( n=200 ) counted for each replicate ( n=3 ) and day across the two parasite populations of α TATLoxP (black) and α TATKO (orange). Mean ± standard error is represented for the respective populations for each day. Dots represent mean percentages of each replicate for the respective populations of α TATLoxP or α TATKO. *t*-test with a Benjamin-Hochberg (BH) correction and a two-way repeated measurements ANOVA were performed: Population *p* = NS (overall potential differences between the population across the days); Day *p* = 0.0128 (variations in the individual populations over the course of the experiment); Interaction *p* = NS (effect of days on the overall difference between the two populations of parasites). (NS: non-significant). **(F)** Isolated α TATKO parasites are deficient in α 1 tubulin K40 acetylation even after several passages in host cells. α TATloxP parasites served as control. No rapamycin was added. Each experiment was performed in three biological replicates.

In *T. gondii*, α TAT is present, and its activity leads to acetylation of SPMTs (Supplementary Figure S4A). This acetylation is modest in the conoid (Supplementary Figure S4B) but more pronounced in developing daughter cells compared to mother cells (Supplementary Figure S4C). Despite the conserved function of α TAT, its presence—but not the acetylation it mediates—has been reported as essential for parasite replication in *T. gondii* 19. To re-examine the essentiality of α TAT, we employed the DiCre conditional system to excise the endogenous locus 24. Since cloning the endogenous Tg α *tat* gene into our GeneSwap vector 5 was unsuccessful, we instead inserted the cDNA of *Pf* α *tat* into a vector containing loxP sites flanking the gene of interest, followed by a fluorescent reporter (YFP) ( α TATLoxP; Supplementary Figure S5A). Upon rapamycin induction, the α *tat* gene was excised, YFP expression was activated, and parasites lost microtubule acetylation (Figure 3 **A**; Supplementary Figure S5B).

The loss of microtubule acetylation occurred within 24 hours of induction and progressively affected nearly 100% of the parasite population over time (Figure 3 **B**). Despite this, the induced parasites continued to grow at rates comparable to non-induced parasites (Figure 3 **C, D**).

To further test whether α TAT-deficient parasites had any fitness disadvantage, we performed a competition assay by mixing equal proportions of WT and α TAT knockout ( α TATKO) parasites. The population ratios remained stable over a 3-week period, with no significant shift detected in either population when measured every two days, indicating that α TATKO parasites do not suffer a growth disadvantage *in vitro* (Figure 3 **E**).

In fact, we successfully cloned α *tat* knockout ( α TATKO) parasites, which could be maintained indefinitely in culture without changes in growth rate, morphology, or the ability to acetylate their microtubules (Figure 3 **F**).

**Figure 4:**
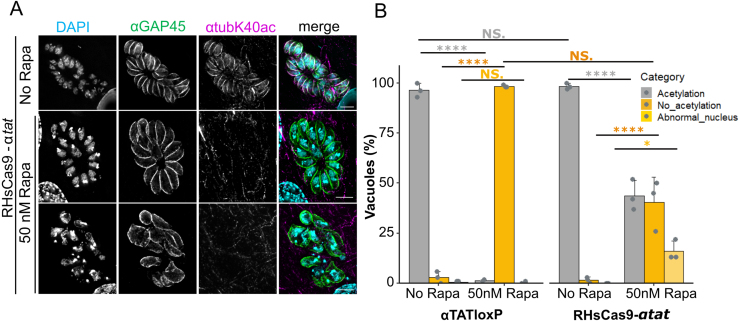
Conditional disruption of α TAT using split-Cas9 leads to loss of α 1 tubulin K40 acetylation and aberrant nuclear morphology in *T. gondii*. **(A)** Loss of α *tat* gene function in split-Cas9 parasites. IFA depicting α 1 tubulin acetylation at K40 in non-induced and induced RHsCas9- α *tat* parasites. Acetylation of α 1 tubulin K40 was lost upon α *tat* gene disruption. In some vacuoles, loss of acetylation was associated with aberrant nuclei and morphology (bottom panel) while other vacuoles appeared healthy (middle panel). Parasites were incubated in the presence or absence of 50 nM rapamycin for 48 h. Scale bars are 5 μ m. **(B)** Loss of K40 acetylation causes aberrant nuclei only in the sCas9 system. Quantification of parasites presenting aberrant nuclei and morphology after α *tat* gene excision in α TATloxP parasites and α *tat* gene disruption in the RHsCas9- α *tat* line. Cells were fixed 48 h post inoculation (p.i.). Data represent three independent experiments. For each condition, 100 vacuoles were counted (total n=300 ). One-way ANOVA followed by Tukey pairwise comparison was performed. *: *p*< 0.05; ****: *p*>0.0001; NS. Non-significant.

These findings contradict the earlier report by Varberg *et al*. (2016) 19, which suggested that disrupting the α *tat* gene impaired parasite replication. Given that Cas9-mediated double-strand breaks are known to cause abnormal parasite morphology 25, we investigated whether these abnormalities might explain the earlier results. Using a conditional split-Cas9 system to disrupt the α *tat* gene, we observed abnormal morphology (Figure 4 **A**). These abnormal parasites could appear in up to 16% of vacuoles in the induced RHsCas9- α *tat* parasites but were absent in induced α TATLoxP parasites upon rapamycin induction (Figure 4 **B**). Furthermore, we successfully isolated RHsCas9- α tatKO clones with confirmed indels in the α *tat* locus (Supplementary Figure S6A). These clones exhibited no additional morphological abnormalities and were stable in culture (Supplementary Figure S6B).

In summary, these results demonstrate that α TAT is responsible for microtubule acetylation in *T. gondii*. However, the absence of α TAT does not affect parasite growth during asexual stages.

### Modification of residues at the position 40 in *T. gondii* do not affect parasite behaviour in asexual stages

Acetylation of K40 in α tubulin has been associated with increased microtubule stability and protection against mechanical stress in other eukaryotes 26, 27. This residue is conserved and acetylated in *T. gondii* α 1 tubulin. To investigate the functional significance of K40 in *T. gondii*, we introduced targeted substitutions at this position: alanine (K40A) and glutamine (K40Q) to prevent acetylation. A methionine (K40M) substitution was inserted to mimic potential methylation, as K40 has been reported to be methylated in another eukaryote 28. Notably, the region surrounding K40 also differs between species: the KTIGGG motif present in *T. gondii* is replaced by the QAGRAN present in *P. berghei* (Table 1). To test whether this alternative sequence affects microtubule function, we replaced KTIGGG with QAGRAN motif in *T. gondii*.

**Figure 5:**
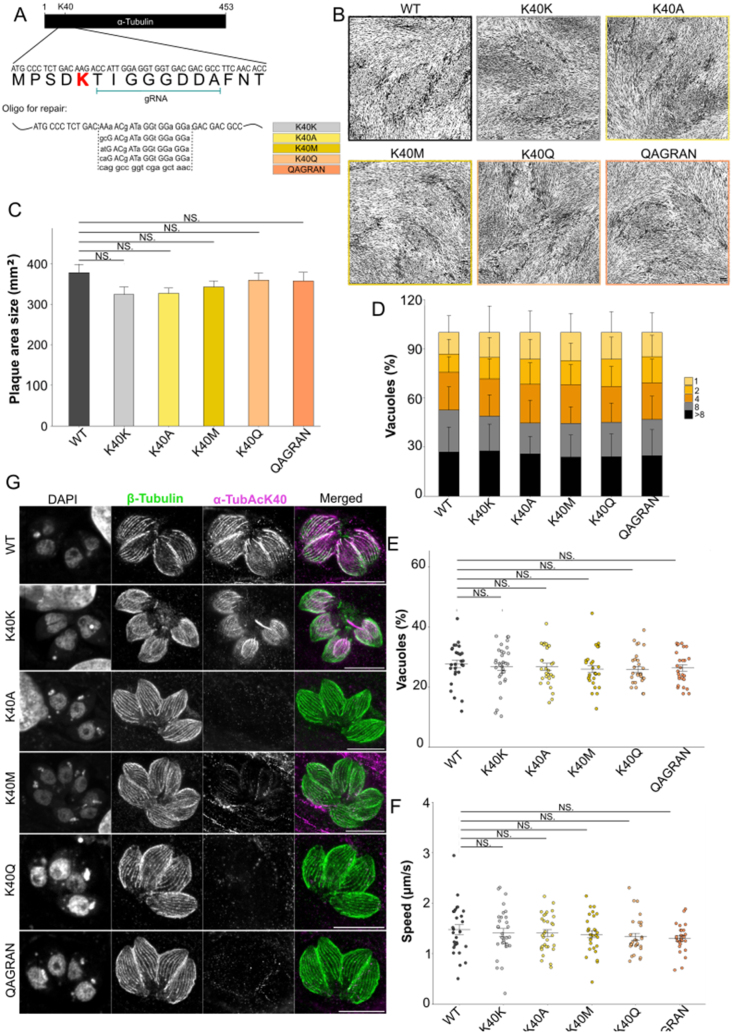
Changes in the lysine 40 in α 1 tubulin has minimal impact on *T. gondii* growth. **(A)** Schematic representation of the strategy followed to create the K40 mutants. Together with the point mutations of the K40 codon, shielding mutations were inserted to avoid continuous cleavage by Cas9. **(B)** Plaque assay resulted in minimal growth defect in parasites with a mutated K40 when compared to the WT. Scale bar: 100 μ m. **(C)** Quantification of plaque area shown in B. Plaque assays were performed in biological triplicates. n=30 per strain. **(D)** Quantification of replication assays showing the percentages of vacuoles containing 1, 2, 4, 8, or more parasites. Assay was performed in biological triplicates n=300 vacuoles. **(E, F)** Gliding assay showed all parasites reach similar distances (E) and same speeds (F). This assay was performed in biological triplicates n=30 parasites. One-tail ANOVA followed by a t Student test was performed for all quantifications shown in this figure. n.s. = non-significant. **(G)** Images depicting the different mutated clones showing the lack of acetylation in microtubules where lysine is not present. Note that the change to methionine shows a minimal acetylation in microtubules.

Using Cas9-mediated site-directed mutagenesis, we introduced these targeted substitutions without incorporating resistance cassettes or significantly altering the locus (Supplementary Figure S6C). We designed a guide RNA targeting the sequence immediately downstream of K40 and provided a 100 base-pair repair oligonucleotide containing the desired mutation along with shielding mutations to prevent continuous Cas9 cleavage (Figure 5 **A**). As a control, we repaired the sequence using an oligo encoding lysine (K40K) along with the shielding mutations. After transfection, clones were screened by PCR to confirm successful mutations (Supplementary Figure S6D).

Structural analysis using AlphaFold did not indicate major changes in the K40 loop upon introducing these mutations, suggesting that the residue remained accessible to α TAT (Supplementary Figure S7). We successfully isolated mutants for all designed variations. All mutant strains were maintained in culture for several weeks without any apparent changes in growth behaviour. In plaque assays measuring lytic capacity, all strains exhibited similar growth rates, with a slight tendency toward slower growth compared to the WT (Figure 5 **B, C**).

Analysis of replication in *T. gondii* mutants carrying K40 substitutions revealed no significant differences in proliferation compared to the WT (Figure 5 **D**). This suggests that α 1 tubulin acetylation is not a major determinant of replication efficiency, aligning with our findings that α TAT is dispensable for parasite survival *in vitro*. While previous studies in other eukaryotes have linked tubulin acetylation to mitotic spindle stability and chromosome segregation 29, 30, our data suggest that *T. gondii* employs alternative mechanisms to ensure proper mitotic progression, likely through microtubule-binding proteins or other post-translational modifications. Furthermore, our analysis of gliding motility showed no significant differences in speed or displacement among the mutants (Figure 5 **E, F**), further supporting that α 1 tubulin acetylation is not a key determinant of *T. gondii* motility. This finding contrasts with studies in mammalian cells, where microtubule acetylation has been linked to enhanced migration and invasion 31.

High-resolution microscopy revealed that all mutants - except for the controls K40K and K40M - lost microtubule acetylation. In parasites with methionine at position 40 (K40M), acetylation levels were weaker than in WT or K40K parasites. Although there are reports of acetylation of N-terminal methionine residues in other proteins 32, acetylation of methionine residues at other positions has not been documented. It is possible that α TAT acetylates any accessible residue within the lumen of microtubules, but methionine is likely less amenable to acetylation, resulting in reduced efficiency and therefore signal (Figure 5 **G**).

Taken together, these data suggest that, in *T. gondii*, the stability and functional integrity of microtubules are primarily maintained through interactions with microtubule-associated proteins and other regulatory factors, rather than being significantly dependent on tubulin acetylation itself.

## DISCUSSION

Microtubule acetylation, particularly at K40, has been widely associated with structural stability, intracellular trafficking, and motility across eukaryotic cells 30, 33–36. However, its role in apicomplexan parasites remains poorly understood. In this study, we explored the presence, localization, and functional significance of microtubule acetylation at position K40 in *P. berghei* and *T. gondii*, highlighting its potential contribution to parasite biology.

*P. berghei* and other rodent-infecting *Plasmodium* species lack α TAT and instead feature a glutamine (Q) at position 40 in both α tubulin isoforms (Table 1), a known mimic of acetylated lysine. Despite this, the introduction of a Q40K mutation in α 1 tubulin in *P. berghei* did not significantly alter parasite growth, infectivity, or motility. This suggests that microtubule acetylation is not essential for *P. berghei*, at least in the asexual and mosquito stages studied. These findings align with previous reports demonstrating that tubulin acetylation is non-essential in other protozoa 37. Additionally, while acetylation has been implicated in facilitating motor protein transport 38, 39, our observations suggest that *P. berghei* parasites do not depend on this mechanism, possibly due to alternative adaptations that compensate for the absence of K40 acetylation. Interestingly, a recent study investigating microtubule inner proteins in *P. berghei* also found no significant differences in infectivity of the parasites lacking these 17. This suggests that the high stability of SPMTs is due to several factors. In fact in *T. gondii*, only the deletion of three microtubule associated proteins showed an impact on microtubules stability 15.

Unlike *P. berghei*, *T. gondii* possesses α TAT, which mediates acetylation in SPMTs and, to a lesser extent, the conoid. However, our conditional knockout studies demonstrated that *T. gondii* can survive and replicate in the absence of α TAT, contradicting previous findings that suggested its essentiality 19. This discrepancy may be attributed to the methodology employed in the earlier study, as we observed morphological defects in parasites subjected to direct Cas9-mediated knockouts but not in our floxed strains upon inducible deletion 25. Our results therefore support the notion that while microtubule acetylation occurs in *T. gondii*, it is not strictly required for parasite survival under *in vitro* conditions.

To further investigate the significance of K40 acetylation, we generated *T. gondii* mutants carrying non-acetylable (K40A, K40Q -acetylation mimic-) or methylation-mimicking (K40M) substitutions. The loss of acetylation in K40A and K40Q mutants, coupled with reduced acetylation in K40M mutants, confirmed that K40 serves as a direct target of α TAT. However, these mutants exhibited only minor effects on replication and no significant differences in gliding motility, reinforcing the notion that K40 acetylation is not crucial for *T. gondii* asexual growth. This aligns with findings in other eukaryotes, where acetylation modulates but does not strictly determine motility 33–35, 40.

While we cannot fully exclude compensatory roles by alternative tubulin isoforms or other regulatory pathways, current evidence suggests that α 2 tubulin does not compensate for the absence of α 1 tubulin acetylation. In *P. berghei*, α 2 tubulin is essential for male gametogenesis, while α 1 tubulin functions predominantly in asexual stages*,* where α 2 tubulin is not expressed*.* Knockouts of either are lethal, indicating distinct and indispensable roles at different stages  9, 22. In *T. gondii*, α 1 tubulin is the dominant isoform in tachyzoites, with other isoforms being structurally divergent and deployed in stage-specific contexts  8, 14. Nevertheless, it remains possible that other post-translational modifications or microtubule-associated proteins maintain microtubule stability and function in the absence of K40 acetylation, highlighting the redundancy and plasticity of the cytoskeletal regulation in these parasites.

The presence of Q40 in *P. berghei* and its lack of α TAT, contrasted with the retention of K40 and α TAT in *T. gondii*, suggests distinct evolutionary trajectories for microtubule regulation in these parasites. Given that motor proteins preferentially interact with acetylated microtubules due to increased binding affinity 30, 38, it is possible that *P. berghei* has evolved alternative post-translational modifications or compensatory mechanisms to support intracellular transport and motility. Additionally, while acetylation plays a role in stabilizing microtubules against mechanical stress in other systems 41, our data suggest that other factors contribute to microtubule stability in apicomplexan parasites.

Together, our findings demonstrate that α 1 tubulin acetylation is not essential for *P. berghei* growth, motility, or infectivity under the tested conditions, and that *T. gondii* can also proliferate in its absence. These results challenge the prevailing assumption that K40 acetylation is universally critical for microtubule function at least in the stages of *P. berghei* and *T. gondii* studied here. Future studies should explore alternative post-translational modifications and their roles in cytoskeletal regulation within apicomplexan parasites. Understanding these mechanisms could provide deeper insights into the evolution of microtubule-associated processes in parasitic protozoa.

## MATERIAL AND METHODS

### Plasmodium berghei methods

#### Ethics statement

All animal experiments were conducted in compliance with the guidelines of the Federation of European Laboratory Animal Science Associations and the Society of Laboratory Animal Science and were approved by the responsible German authorities (Regierungspräsidium Karlsruhe, Germany). Mice were obtained from Janvier or Charles River Laboratories and kept in the dedicated animal facility of the University of Heidelberg following current guidelines, with three mice per cage, access to food and water *ad libitum*, and environmentally enriched housing conditions.

#### Animals and parasites

Parasite lines were generated using the parental *P. berghei* ANKA strain 42 or the *P. berghei* Cas9 strain (generously gifted by Ellen Bushell, Umeå university, Sweden). These lines were produced using 4-6-week-old female Swiss CD1 or NMRI mice obtained from Janvier Labs (Le Genest-Saint-Isle, France). Swiss CD1 or NMRI mice were also used for parasite propagation and for infecting *A. stephensi* mosquitos. For transmission experiments assessing sporozoite infectivity, 4-6-week-old female C57/BL6 mice from Charles River laboratories (Sulzfeld, Germany) were used. *A. stephensi* mosquitos were bred and maintained following standard procedures.

### Generation of the *P. berghei* Q40K mutant via double homologous cross over recombination

The single-point mutant was initially generated through classical double homologous cross over recombination. Mutant generation was performed sequentially, beginning with a positive selection step followed by negative selection. First, the open reading frame (ORF) of α 1 tubulin was replaced by a codon-modified Q40Kcm variant, combined with a selection cassette containing both positive and negative selection markers. Therefore, the linearized vector was transfected into the parental *P. berghei* strain (PbANKA) using standard protocols 43. Transgenic parasites were selected via pyrimethamine (0.07 mg/mL, positive selection) that was administered via the drinking water of the mice. As this gives rise to a mixed population, positive selection was followed by limiting dilution to obtain isogenic parasites. For limiting dilution, 20 NMRI mice (Janvier labs, Le Genest-Saint-Isle, France) were each injected intravenously with statistically a single-blood stage positive parasite. Subsequently, both the selection cassette and the codon-modified α 1 tubulin ORF were excised using negative selection with 5-fluorocytosine (5-FC, 1.5 mg/mL). Parasites still harbouring the selection cassette express the yeast enzyme cytosine deaminase and uridyl phosphoribosyl transferase (yFCU) that metabolizes the prodrug 5-FC into its toxic analogue 5-fluorouracil. As a result, only transgenic parasites that successfully excised the selection cassette and thereby lack the yFCU enzyme, survived negative selection as they become insensitive to 5-FC. The initial construct was designed as such that upon negative selection, both the selection cassette and the introduced codon-modified α 1 tubulin ORF are recycled. To generate the final mutant, transgenic parasites were provided with a linearized vector containing the α 1 tubulin ORF harbouring the Q40K single-point mutation without codon modification. These parasites were subjected to 5-FC-based negative selection, leading to the integration of the non-codon-modified Q40K ORF into the endogenous locus. As before, negative selection was followed by limiting dilution to isolate isogenic parasite clones. Once mice reached a parasitemia of 1–3%, mice were anesthetized (120 mg/kg ketamine, 16 mg/kg xylazine, intraperitoneally) and blood was collected via cardiac puncture. Transgenic parasites were stored as frozen cryo-stabilates.

### Generation of the *P. berghei* Q40K mutant via CRISPR/Cas9

As the generation of a single-point mutation mutant using classical double homologous crossover recombination revealed to be very challenging and time consuming, an alternative approach using CRISPR/Cas9 was used. For this strategy, a constitutively expressing Cas9 parasite line (PbA Cas9), generously provided by Ellen Bushell, Umeå university, Sweden, was used as parental line and mutant parasite generation was done as previously published 44 with the following modifications: Single-guide RNAs (sgRNAs), required for site-specific Cas9 activity, were episomally expressed using a modified version of the pPbHIT (PbCS-HIT) vector, also kindly gifted by Ellen Bushell (Umeå university, Sweden). Modifications to the vector included the removal of the hsp70 3’ UTR via Esp3I and AatII restriction sites.

To achieve high editing efficiency, sgRNA target sites were selected within 200 bp up- or downstream of the desired mutation site. sgRNAs were designed using the Eukaryotic pathogen CRISPR guide RNA design tool (PlasmoDB-28 as *P. berghei* reference genome), excluding off-target binding. Candidate sgRNAs were prioritized based on proximity to the targeted mutation site, Doench-Root efficiency scores surpassing 5099 and uniqueness within the genome. Preferential consideration was given to sgRNA sequences overlapping with the mutation target site, as the introduced mutation would confer resistance to re-cleavage by Cas9. Sequences of all sgRNAs, including the respective overhangs for ligation, are listed in Supplementary Table S1A.

As the Cas9 endonuclease introduces DNA double strand breaks, tailored repair templates were supplied to facilitate homology-directed repair. Two different approaches – single-stranded (ss) and double-stranded (ds), were used. The ss template consisted of a 198 bp DNA fragment complementary to the target region, encoding both the desired single-point mutation and up to four silent shielding mutations to prevent Cas9 re-cleavage (Figure 2 **A**). For the ds template design, homologous regions of 400–500 bp up- and downstream of the mutation site were generated. These regions were amplified via a two-step extension overlapping PCR, using primers that introduced both the single-point mutation and shielding mutations within the sgRNA seeding region.

For transfection, equimolar amounts (up to 5 μ g) of either ss or ds linearized repair template DNA and plasmid DNA were co-precipitated and transfected into parasites as previously described 43. One day post transfection, mice were provided with drinking water containing 7 mg/mL pyrimethamine for five days, after which mice were provided again with normal drinking water. Parasitaemia was monitored daily through Giemsa-stained blood smears. Once parasitaemia exceeded 1%, mice were bled and parasites were isolated and genotyped to confirm successful transfection. Similarly, transfection was followed by limiting dilution to obtain isogenic parasites. For this, six to eight Swiss CD1 mice (Janvier Labs, Le Genest-Saint-Isle, France) were each injected intravenously with a single-blood stage positive parasite. Isogenicity was verified via genotyping PCR and sequencing.

### Immunofluorescence assay (IFA) of *Plasmodium*

For IFA of blood-stage parasites, cells were fixed in 4% PFA in PBS (v/v). Fixation was carried out for 30 minutes at 37 ${}^{\circ }$ C with agitation, followed by centrifugation at 3000 g for 2 minutes. After fixing, the cells were quenched in permeabilization/quenching buffer for 15 minutes, then washed twice with 1% BSA in PBS (w/v). Cells were blocked in 3% BSA in PBS (w/v) for 1 hour at room temperature with agitation. Following another centrifugation step (2 minutes at 3000 g), cells were incubated with primary antibodies in a total volume of 100–300 μ L for 12–24 hours at 4 ${}^{\circ }$ C with agitation. The cells were then washed three times with 1% BSA in PBS (w/v) for 10 minutes each before staining with secondary antibodies for 1–2 hours at room temperature with agitation. The antibodies and dyes used are listed in Supplementary table S1B. Subsequently, cells were washed three more times in 1% BSA in PBS (w/v) for 10 minutes each, with Hoechst added during the second wash for nuclear staining. IFA samples were stored in PBS at 4 ${}^{\circ }$ C until ready for imaging. Prior to imaging, cells were pelleted for 2 minutes at 3000 g, and 3–5 μ L of resuspended cells were transferred to a glass slide, covered with a coverslip, and sealed with wax. Image acquisition was done at a Zeiss Airyscan 2 LSM900 laser scanning confocal microscope using a 63x oil objective and Z-stack acquisition. Images were 3D processed by the internal 3D Airyscan processing option using the Zeiss ZEN 3.1 software and further processed and analysed using Fiji.

#### Determination of the asexual blood stage growth rate

The growth rate of asexual blood stages was evaluated in mice that had been previously injected with a single infected red blood cell (iRBC) during a limiting dilution. Parasitaemia levels were measured on day 8 post-infection in mice confirmed to harbour isogenic parasites with the correct genotype. These parasitaemia values were then used to retrospectively calculate the growth rate, as demonstrated in the formula below. The asexual growth rate is expressed as a fold increase per 24 hours 45, 46:

$$\frac{\frac{\text{parasitemia (\%)}}{100}\times 7\times 10^{6}\;\text{RBCs}/\mu l^{6}\times 2000\;\mu l^{*}}{\text{number of parasites injected}}$$

*The total blood volume of a 6–8-week-old Swiss mouse is estimated to be approximately 2000 μ l, with each μ l of blood containing around 7 × 10 ${}^{6}$ RBCs 47.

#### Mosquito infection

Two naïve Swiss mice were injected with 20 × 10 ${}^{6}$ iRBCs intraperitoneal (i.p.). Three days post infection, the presence of male gametocytes was assessed by determining exflagellation. For this, a drop of tail blood was placed on a glass slide, covered with a coverslip and examined 12 minutes later. Exflagellation centres were observed in multiple fields of view with similar erythrocyte densities using an Axiostar Plus (Carl Zeiss, Jena, Germany) equipped with a 40x objective and a phase contrast ring. If at least 3–4 exflagellation events per field of view were detected, mice were anesthetised with 100 mg/kg ketamine and 3 mg/mL xylazine i.p. Approximately 200 female *Anopheles stephensi* mosquitos were then allowed to feed on the infected mice for around 20 to 40 minutes. Infected mosquitos were kept at 21 ${}^{\circ }$ C and 80 % humidity and provided with both sugar and salt pads.

#### Analysis of midgut infection and oocyst development

To check whether parasites can infect mosquito midguts and develop oocysts, mosquito midguts were dissected on two separate days between day 11 and day 14 post infectious blood meal. Midguts were dissected in 100 μ l PBS on ice. After permeabilization for 20 minutes on RT in 1% Nonidet-P40 (A1594, AppliChem GmbH, Darmstadt, Germany), midguts were stained for 30 minutes to 1.5 hours with 0.1% mercurochrome NF XIII (M7011-25G, Sigma-Aldrich, St. Louis, MO, USA). After staining, midguts were washed thrice with PBS. The stained midguts were placed on a glass slide, covered with a cover glass and imaged at a Widefield Zeiss microscope (Axiovert 200 M, Carl Zeiss, Jena, Germany) at 10x magnification (NA 0.5) using a green filter. Both the infection rate (number of infected midguts in total midguts) and the oocyst count per infected midgut were determined.

#### Analysis of sporozoite formation

To test whether parasites can form sporozoites, the number of sporozoites in both midgut oocysts and salivary glands were determined. For this, midguts and salivary glands from the same mosquitoes were dissected on two separate days between day 17 to day 22 post infection. Both midguts and salivary glands were dissected into two separate tubes containing 50 μ l PBS on ice. Sporozoites were released by crushing each tissue for 1 minute with a pestle. From the sporozoite suspension, 10 μ l were placed onto a Neubauer counting chamber and sporozoites were counted at a Zeiss Axiostar Plus Light microscope at 40x magnification using a phase contrast ring.

#### *In vitro* sporozoite gliding assays

To determine whether sporozoites are motile, an *in vitro* gliding assay was performed. For this, salivary glands were dissected into 50 μ l RPMI and sporozoites were released as described above. The sporozoite suspension was filled up with RPMI to 1 mL and the suspension was carefully underlaid with 17 %ccudenz/dH ${}_{2}$ O (AN7050, Accurate Chemical & Scientific Corp., Westbury, NY, USA). Centrifugation for 20 minutes at 2800 rpm at RT (without break) separated sporozoites from cell debris. The sporozoite containing interphase was carefully collected using a Pasteur pipette (1.4 mL) and transferred into a 1.5 mL tube. Sporozoites were pelleted for 3 minutes at 13000 rpm. The supernatant was removed and sporozoites were activated in 3 % BSA/RPMI (Carl Roth GmbH + Co. KG), transferred to a 96-well optical glass bottom plate (Thermo Fisher Scientific, Waltam, MA, USA) and spun down for 3 minutes at 1000 rpm at RT. Within 1-hour post activation, sporozoite gliding was observed taking 5 minutes movies at a Widefield Zeiss microscope (Axiovert 200M, Carl Zeiss, Jena, Germany) at 25x magnification (NA 0.8, oil) in DIC with a frame rate of 3 s/frame.

Sporozoite gliding was analysed single-blinded and both the gliding pattern as well as the speed was determined. Gliding patterns were categorized as following: Continuous movers moved for at least 50 frames without stopping more than 10 frames and being constantly attached to the substrate. Partial movers moved more than 1 parasite length but less than 50 frames. Non-productive movers comprised parasites that either stayed completely attached and moved less than 1 parasite length, parasites that patch glided by moving back and forth for less than 1 parasite length, parasites that waved by staying attached at one side only, and parasites that floated in the medium. The speed was then determined from all sporozoite classified as continuous movers. For this, movies were Z-projected using Fiji and the circle diameter was measured. From the circle diameter and the number of circles moved, the speed can be deduced.

#### Transmission to mice

To check whether parasites can infect mice, a transmission assay was carried out. Testing for natural transmission via mosquito bites, 10 infected mosquitoes were put into a cup and allowed to feed for 10–20 minutes on a naïve C57/BL6 mouse until at least 6 out of 10 mosquitos have fed. In parallel, salivary gland sporozoites were isolated as described before and 1000 sporozoites were injected intravenously into the teil vein of a naïve C57/BL6 mouse. For each biological replicate 3–4 C57/BL6 mice (6–8 weeks old, Charles River Laboratories, Sulzfeld, Germany) were used. Starting from day 3 post infection, the parasitemia was determined daily. Mice having reached parasitemias between 1–2% were sacrificed by cervical dislocation.

### *Toxoplasma gondii* methods

#### Plasmid construction

##### Generation of Tg α TAT mutant strain

The construct Tg α *tat ko*-construct was generated to delete the α *tat* gene in *T. gondii* RH Δ Ku80 parasites. For this purpose, parts of the 5 ${}^{\prime }$ and the 3 ${}^{\prime }$ UTR of Tg α *tat* (TGME49_319600) were amplified by applying the primers 1596/1597 (1184 bp) and 1598/1599 (1117 bp), respectively. The primers attached restriction sites to the amplified DNA (5 ${}^{\prime }$ UTR: ApaI, EcoRI; 3 ${}^{\prime }$ UTR: AscI, XmaI). The restriction sites were exploited for integrating the 5 ${}^{\prime }$ and 3 ${}^{\prime }$ UTR into the parental vector resulting in the Tg α *tat* ko-construct (9422 bp). The construct Tg α *tatGeneSwap*-construct was created by replacing the KillerRed with the Pf α *tat* cDNA in the Tg α *tat ko*-construct. Synthesised Pf α *tat* cDNA (PF3D7_0924900) served as PCR template. By using the primers 1849/1850 the restriction sites XbaI and BglII were attached to the Pf α *tat* cDNA (581 bp). To insert the Pf α *tat* cDNA into the Tg α *tat* ko-construct, the cDNA was restricted with XbaI/ BglII while the Tg α *tat* ko-construct was digested with AvrII/ BglII. The enzyme AvrII creates 5 ${}^{\prime }$ extensions that can be ligated to overhangs created by XbaI. The subsequent ligation reaction resulted in the Tg α *tat GeneSwap*-construct (9233 bp).

##### α Tubulin K40 mutants

The plasmids used for the generation of the various α tubulin K40 mutant strains through a transient CRISPR-Cas9 system in this study comprised of a Cas9 construct to create the precise, targeted point mutations. The Cas9 construct was YFP tagged to facilitate the selection of positive clones post transfection using FACS sorting. The sgRNAs to yield the various mutants was generated through annealing oligos 8165 and 8166 at 95°C followed by insertion into the BsaI cut site of the Cas9-YFP vector 48.

All oligos are detailed in Supplementary Table S1C.

#### Culture conditions

Human foreskin fibroblasts (HFF) were cultured in Dulbecco’s modified Eagle’s medium (DMEM) supplemented with 10% foetal bovine serum, 25 mg/mL gentamicin, and 2 mM L-glutamine until they reached 100% confluency. Parasites were then allowed to infect the HFF monolayer and maintained at 37 ${}^{\circ }$ C with 5% CO ${}_{2}$ in an incubator.

#### Transfection in *Toxoplasma gondii*

##### Generation of Tg α TAT mutant strain

For transfection purposes, 60 μ g of Tg α TATKo construct (linearized with ApaI) or 60 μ g of Pf α TATGeneswap vector (linearized with ApaI and ApaLI) were ethanol-precipitated. For this purpose, 2.5 volumes of ice-cold 100% ethanol and 1/10 NaAc (3 M, pH 5) were added to the restriction reaction mix. The mix was incubated at -20 ${}^{\circ }$ C for at least 14 h. After this, DNA was pellet for 60 minutes at 4 ${}^{\circ }$ C with maximum speed. Two washing steps (centrifugation: 10 minutes, 4 ${}^{\circ }$ C, maximum speed) with ice-cold 70% ethanol followed the first centrifugation step. The supernatant was taken off under sterile conditions and the pellet was air dried for approximately 15–30 minutes. The pellet was then resuspended in 100 μ l of cytomix (electroporation buffer: 10 mM K ${}_{2}$ HPO ${}_{4}$ /KH ${}_{2}$ PO ${}_{4}$ , 25 mM HEPES and 2 mM EGTA pH 7.6, 120 mM KCl, 0.15 mM CaCl ${}_{2}$ , 5 mM MgCl ${}_{2}$ with 5 mM KOH adjusted to pH 7.6).

Freshly lysed parasites were spun down at 1200 rpm for 10 minutes at room temperature. The parasite pellet was resuspended in 640 μ l cytomix together with 30 μ l ATP (100 mM), 30 μ l GSH (100 mM) and 100 μ l of linearized plasmid DNA were transferred to an electroporation cuvette. Electroporation settings were chosen as follows: 1700 V, 2 pulses for 0.2 seconds. After this procedure, the parasites were transferred onto confluent human fibroblast foreskin (HFF) cells in a 6 cm dish. Selection for stable clones was achieved by adding the selection markers Mycophenolic acid (MPA, 25 mg/mL) and Xanthine (XAN, 40 mg/mL) to the parasites 17–24 hours after transfection. The genomic DNA from the single clones was extracted and subjected to PCR (primers in Supplementary Table S1C) to identify positive clones (Supplementary Figure 5B).

To obtain a clean knockout strain ( α TATKO), the α TATLoxP strain was induced using 50nM rapamycin for 48 hours, followed by a serial dilution in a 96-well plate while maintaining the parasites under rapamycin selection. After 7 days, single plaques were isolated to obtain a single α TATKO to perform the competition assay. The KO clone was verified through the observation of GFP-positive parasites in an IFA and PCR (Supplementary Figure 5B).

##### Generation of α tubulin k40 mutants

To generate the different point mutations for the lysine 40 (K40) in the *T. gondii* α 1 tubulin, 10 μ g of the Cas9-YFP plasmid with the repair templates were ethanol-precipitated and pelleted as previously described. The DNA pellet was then resuspended in 100 μ L of Lonza P3 Primary cell transfection buffer. Freshly lysed RH Δ Ku80 DiCre parasites were pelleted at 1500 g for 5 minutes, mixed with the DNA, and transfected using the FI-158 program in the Amaxa 4D Nucleofector system. The parasites were transferred to the HFF monolayer for recovery. After 24–48 hours, the parasites were FACS sorted into 96 well plates (5/10 events per well). Parasites were kept undisturbed for 5 days until they would form single plaques. The genomic DNA from the single clones was extracted and subjected to PCR using primers 8175/8177 (1045 bp) to identify clones lacking amplification, indicative of successful editing (Supplementary Figure 6D). The positive clones confirmed through the verification PCR were then subjected to a PCR amplification using primers 8176/8177 (1509 bp) to generate DNA fragments foe the identification of the various point mutations through Sanger sequencing.

#### Immunofluorescence assay (IFA) in *Toxoplasma*

HFFs were cultured on sterile coverslips in 24-well plates to establish a monolayer for parasite infection. The parasites were then allowed to infect the HFF monolayer for 24–48 hours. The coverslips were fixed with 4% Paraformaldehyde (PFA) for 20 minutes followed by block-permeabilisation using 3% BSA and 0.1% Triton-100 in PBS for 20 minutes. Primary and secondary antibodies of required dilutions were prepared in the block-permeabilising solution (See antibody list in Supplementary Table S1D). The coverslips were mounted onto slides using ProLong™ Gold Antifade Mountant with DNA Stain DAPI (Thermo Scientific, USA) and imaged using a Leica DMi8 inverted live cell widefield microscope or an Abberior STED microscope.

#### Plaque assay

Parasites were released by passing them through a 26G needle. 1000 parasites per well were allowed to infect a HFF monolayer cultured on a 6-well plate. The parasites were incubated, left undisturbed, for 5–7 days to form plaques. The cells are then washed with PBS and fixed with ice-cold 100% Methanol (−20 ${}^{\circ }$ C) for 20 minutes and later stained with Giemsa stain to visualise the plaques. The plaques were imaged using a Leica DMi8 inverted live cell widefield microscope. Ten random plaques were then selected, and their respective plaque area sizes were determined using ImageJ. The mean plaque area size and standard error mean (SEM) were quantified.

#### Replication assay

Parasites were allowed to infect HFF monolayer seeded glass coverslips for one hour. The non-invaded parasites were then washed off with PBS to synchronise the replication of parasites within the host cells. DMEM media (Sigma) was added to the coverslips and the parasites were incubated at 37°C for 24 hours to allow for replication within the host cells. The coverslips were then fixed, and the standard IFA protocol was followed as described previously using anti-GAP45 (1:5000), to visualise the parasites, as the primary antibody. The parasites were imaged using the Leica DMi8 inverted live cell widefield microscope (Leica, Germany) and the number of vacuoles in the various cell stages of the parasites was determined for each mutant strain. To improve the statistical significance of the observations, three biological replicates were performed.

#### Live Gliding assay

Pre-warmed FBS was added onto the surface of Cellvis 29 mm glass bottom dish (Cellvis, USA) and incubated for 1–2 hours and then aspirated to provide a surface to facilitate parasite gliding. Parasites were harvested and passed through a 3 μ m filter to remove debris. The parasites were pelleted at 1500 g for 5 minutes and washed with a pre-warmed gliding buffer to remove media. They were then resuspended in the gliding buffer (1 mM EGTA and 100 mM HEPES in HBSS solution) ensuring a concentration of 4 × 10 ${}^{6}$ parasites/mL. The parasites were added to the FBS-coated glass bottom dishes, and the gliding efficiency was recorded using the Leica DMi8 inverted live cell widefield microscope by capturing the gliding of the parasite every second for a total of 10 minutes in the DIC channel. The distance travelled by the parasites ( ∼10 parasites per replicate) as well as the speed at which the gliding motion occurred were assessed using the ManualTracking Plugin in Image J across three independent biological replicates.

#### α TAT competition assay

5 × 10 ${}^{5}$ parasites of α TATLoxP and α TATKO strains, respectively, were counted and mixed in a 6 cm dish to obtain a final count of 1 × 10 ${}^{6}$ parasites. Roughly 10 ${}^{6}$ parasites were passed every 2 days onto new cells for a duration of 24 days. The composition of α TATLoxP and α TATKO (GFP positive) parasites was also estimated every 2 days in an IFA using anti-GFP (1:500, Roche) and anti-GAP45(1:10000) antibodies. The coverslips were imaged using a Leica Widefield microscope, and the images were processed using ImageJ. The percentages of vacuoles with GFP-positive ( α TATKO, “Green”) and GFP-negative ( α TAT, “Non-green”) parasites were quantified. The mean, standard error, and statistical significance were estimated using RStudio. The potential variations in the means of α TATLoxP and α TATKO parasites across 3 biological replicates for the respective days were evaluated using a two-sample t-test with a Benjamin-Hochberg (BH) correction to eliminate the occurrence of false positives. To estimate the global variations in the two populations across the various days, a two-way repeated measures ANOVA was performed. This test evaluated the overall potential differences between the population across the days (Population *p*= NS), variations in the individual populations over the course of the experiment (Day *p*=0.0128), and the effect of days on the overall difference between the two populations of parasites (Interaction *p*= NS). This assay was performed in biological triplicate.

#### Software and online applications

All sequences were obtained from the ToxoDB and PlasmoDB databases 49, 50; Release 68, 7 May (2024). Protein structure predictions and models were generated using ColabFold v1.5.2 51, Alphafold2 52 and 3 53, PyMol (Schrodinger, LLC. 2010. The PyMOL Molecular Graphics System, Version 2.5.2.) and ChimeraX (v. 1.9; 54). sgRNA designing was aided by the Eukaryotic Pathogen sgRNA Design Tool (EuPaGDT) 55. *In silico* cloning was conducted using ApE (v. 3.1.3; 56) and Benchling (https://benchling.com). BioEdit (v.7.0.5.3; 57) and Clustal Omega (https://www.ebi.ac.uk/jdispatcher/msa/clustalo) was used in the analysis of sequencing results. Image acquisition was carried out using Leica LAS X software (v. 3.10.0.28982; RRID:SCR_013673) for Leica DMi8 inverted live cell widefield microscope, Zen v2.6 for Image acquisition at Zeiss Axiovert 200M, Zen v3.1 for Image acquisition at Zeiss LSM 900 Airy Scan2 and Imspector (v.16.3.19714-w2408) for the Abberior STED microscope. All image processing and analysis were done using ImageJ (v. 2.16.0/1.5p) 58. Inkscape v1.3 and v1.4 was employed to generate the figures for this manuscript (www.inkscape.org).

GraphPad prism, 6 (GraphPad Software, San Diego, CA, USA; https://www.graphpad.com) and RStudio (v. 4.4.1) was utilised to perform statistical analysis and data visualisations. Two-tailed Student t-test and one-way ANOVA were used to determine the statistical significance.

## AUTHORS CONTRIBUTIONS

Research design: AMB, BS, FF, MM and EJR. Performing of experiments: TK, KR, JFS, BS and MK. Data analysis: TK, KR, JFS and MK. Manuscript writing: TK, MR, JFS, AMB, FF, MM and EJR. Resources contribution: FF, MM and EJR.

## SUPPLEMENTAL MATERIAL

All supplemental data for this article are available online at www.microbialcell.com. 



## CONFLICT OF INTEREST

The authors declare no conflict of interest.

## ABBREVIATIONS

SPMTs – subpellicular microtubules

TAT – tubulin acetyltransferase

WT – wildtype

KO – knockout
