# Supplementary material for: Luminal acetylation of microtubules is not essential for Plasmodium berghei and Toxoplasma gondii survival
Source: Microb Cell. 2025 Dec 17;12:299–313. doi: 10.15698/mic2025.12.863 (PMC12795560; doi:10.15698/mic2025.12.863)
Supplement: Supplementary file 1 [file mic-12-299-s01.pdf]

Supplementary Figures

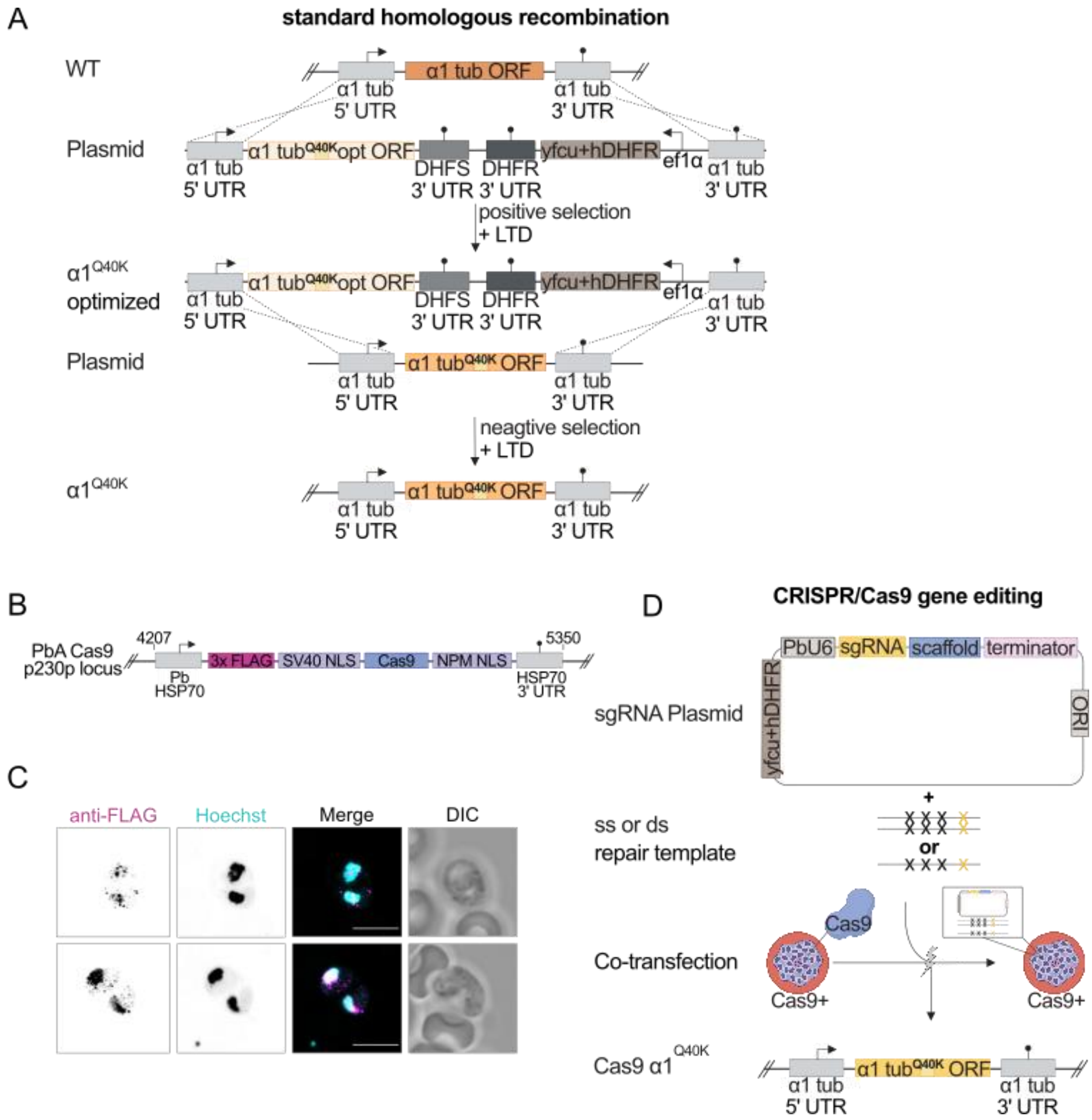

Supplementary Figure 1: Schematic overview of the genomic locus of the α1 tubulin mutants in *P. berghei*. A) The mutant parasite line $\alpha1^{Q40K}$ was generated via standard homologous recombination. **B)** Schematic representation of the PbCas9 p230p integration locus. The Cas9 endonuclease is driven by the PbHSP70 promotor, flanked by two nuclear localisation signals (NLS), and tagged with a 3x FLAG tag. The Cas9 integration site into the p230p locus spans from base pair position 4207-5350 within the locus as indicated with numbers above the scheme. Scheme is not drawn to scale. **C)** Detection of nuclear localization of Cas9 by immunofluorescence in asexual blood stages using an anti-FLAG antibody. FLAG tag in magenta, Hoechst in cyan, scale bar: 5 µm. **D)** Strategy to generate the mutant parasite line Cas9 $\alpha1^{Q40K}$ via CRISPR/Cas9 gene editing. UTR: untranslated region; SV40 NLS: simian virus 40 nuclear localization signal; NPM NLS: nucleophosmin nuclear localization signal; ORF: open reading frame; opt- optimized; DHFS: dihydrofolate synthase; DHFR: dihydrofolate reductase; yfcu: uridyl phosphoribosyl transferase; hDHFR: human dihydrofolate reductase; LTD: limiting dilution; sgRNA: single guide RNA; ORI: origin of replication; ss: single strand; ds: double strand.

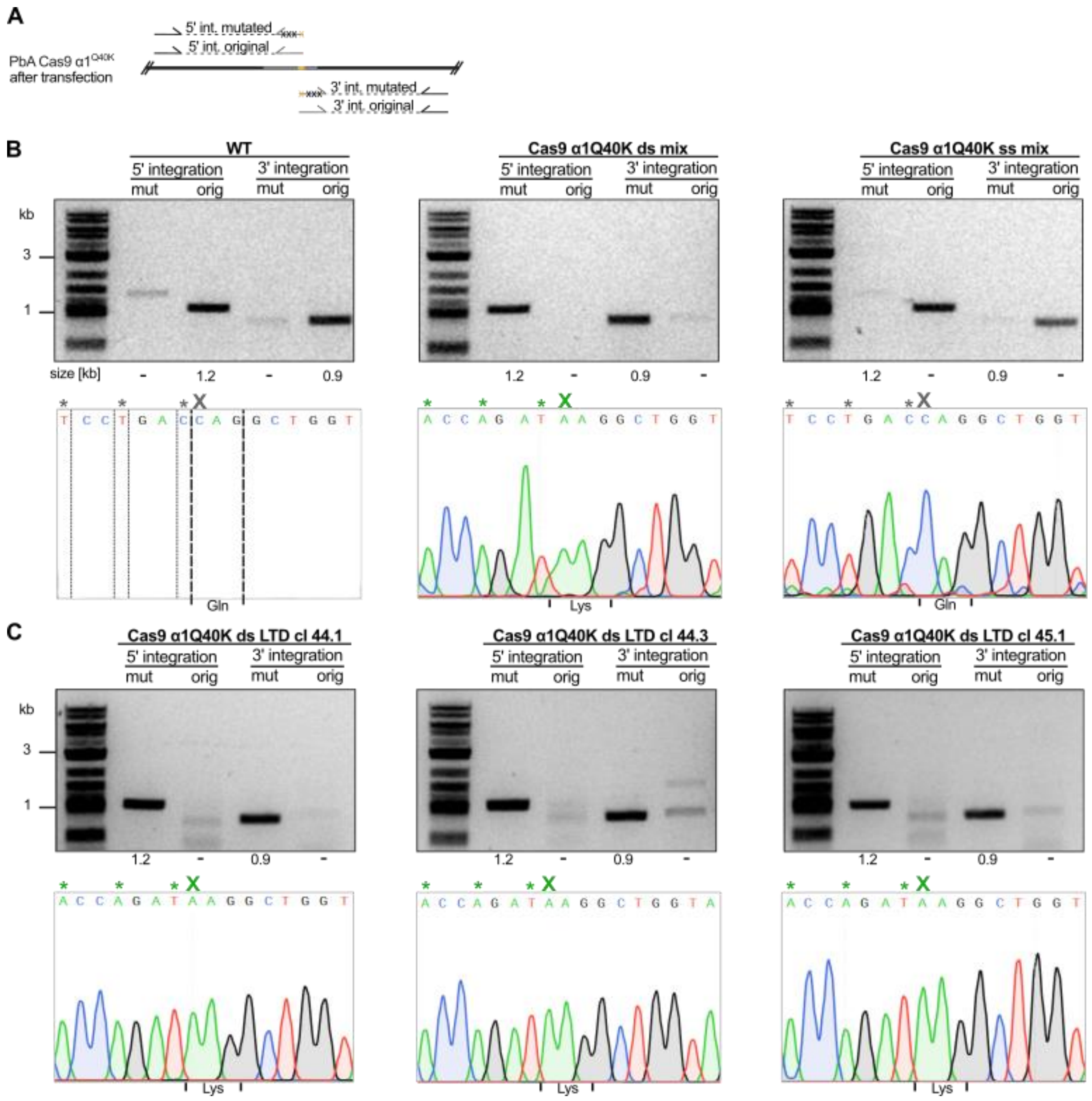

Supplemental figure 2: Generation of an alpha 1 tubulin Q40K *P. berghei* mutant using CRISPR/Cas9. A) Schematic representation of the genotyping approach used to confirm the insertion of the Q40K point mutation. Grey and yellow crosses indicate silent shielding mutations and the desired point mutation, respectively. Two distinct primer pairs were employed for 5' and 3' integration verification. Schematic primer locations are indicated. **B, C)** Genotyping PCRs and sequencing results of (B) WT parasites, as well as parasites transfected with either the double-stranded (ds) or single-stranded (ss) repair templates, and in (C) clonal parasite lines after limiting dilution (LTD) with specific clone numbers (cl). Both 5' and 3' integration was confirmed using primer sets designed to bind either in the non-mutated, original WT locus (orig) or in the mutated locus (mut). Expected fragment sizes are shown below the gel images in kilobases (kb). Sequencing results in the form of chromatograms, along with their nucleotide sequences, are presented below the respective genotyping gel image. Grey symbols above the chromatograms represent WT-like sequences, while green symbols indicate correct integration of both shielding mutations (*) and the targeted mutation (X). Dotted lines mark the expected mutation positions. The three-letter amino acid code of the targeted codon is shown below the chromatogram.

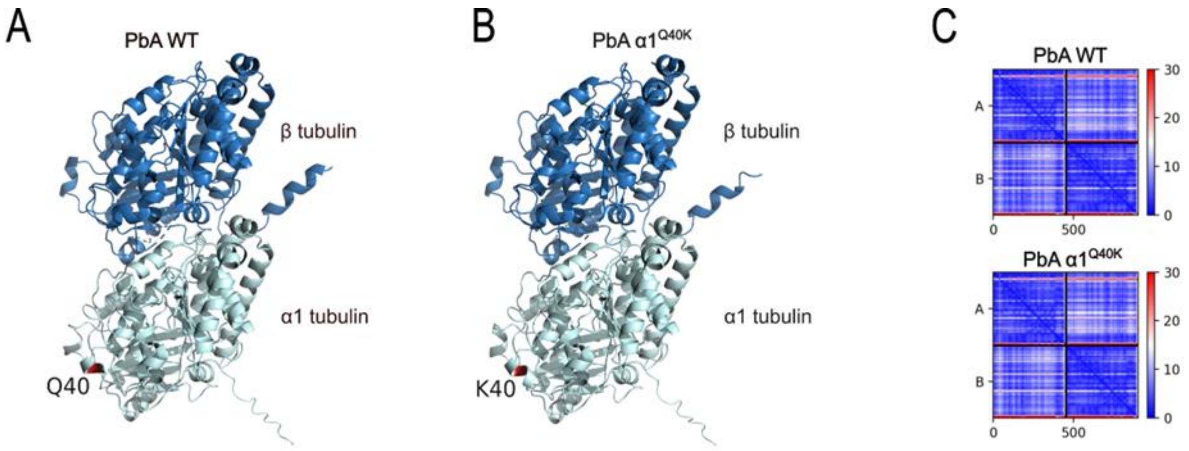

Supplemental Figure 3: Protein structure predictions of Q40K mutated α1 andβ tubulin in *P. berghei*. **(A)** Protein structure prediction of the *P. berghei* WT (WT) tubulin dimer consisting of the β tubulin variant and the α1 tubulin variant. Amino acid position 40 with its residue glutamine (Q) is indicated in red. **(B)** Protein structure prediction of the *P. berghei* alpha 1 Q40K ($\alpha1^{Q40K}$) mutated tubulin dimer. β tubulin variant and mutated α1 tubulin variant. Amino acid position 40 with its mutated residue lysine (K) is indicated in red. **(C)** Alignment and PAE score of both protein structures shown in A (WT) and B($\alpha1^{Q40K}$ mutant).

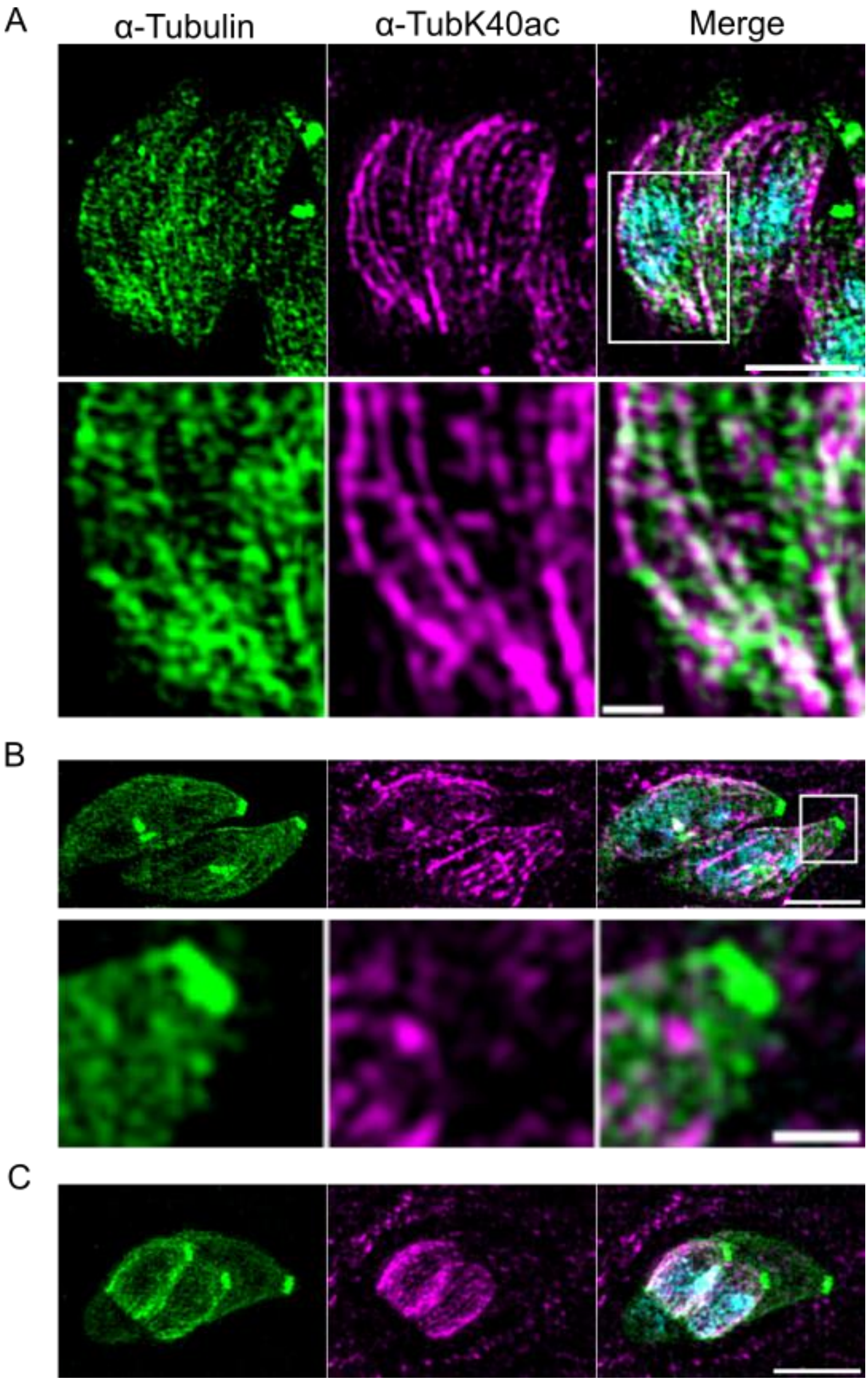

Supplementary Figure 4: Super-resolution microscopy depicting acetylation of α1 tubulin lysine 40 (α-tubK40ac) in *T. gondii* during asexual replication. Using IFA, YFP-α1 tubulin (in green) expressing RH parasites were fixed with 4% PFA and stained for K40ac α1 tubulin (in magenta) exploiting the antibody 6-11 B-1 which specifically recognizes this PTM **(A, B, C)**. White squares indicate enlarged areas underneath the respective pictures. Scale bars: 2.5 µm

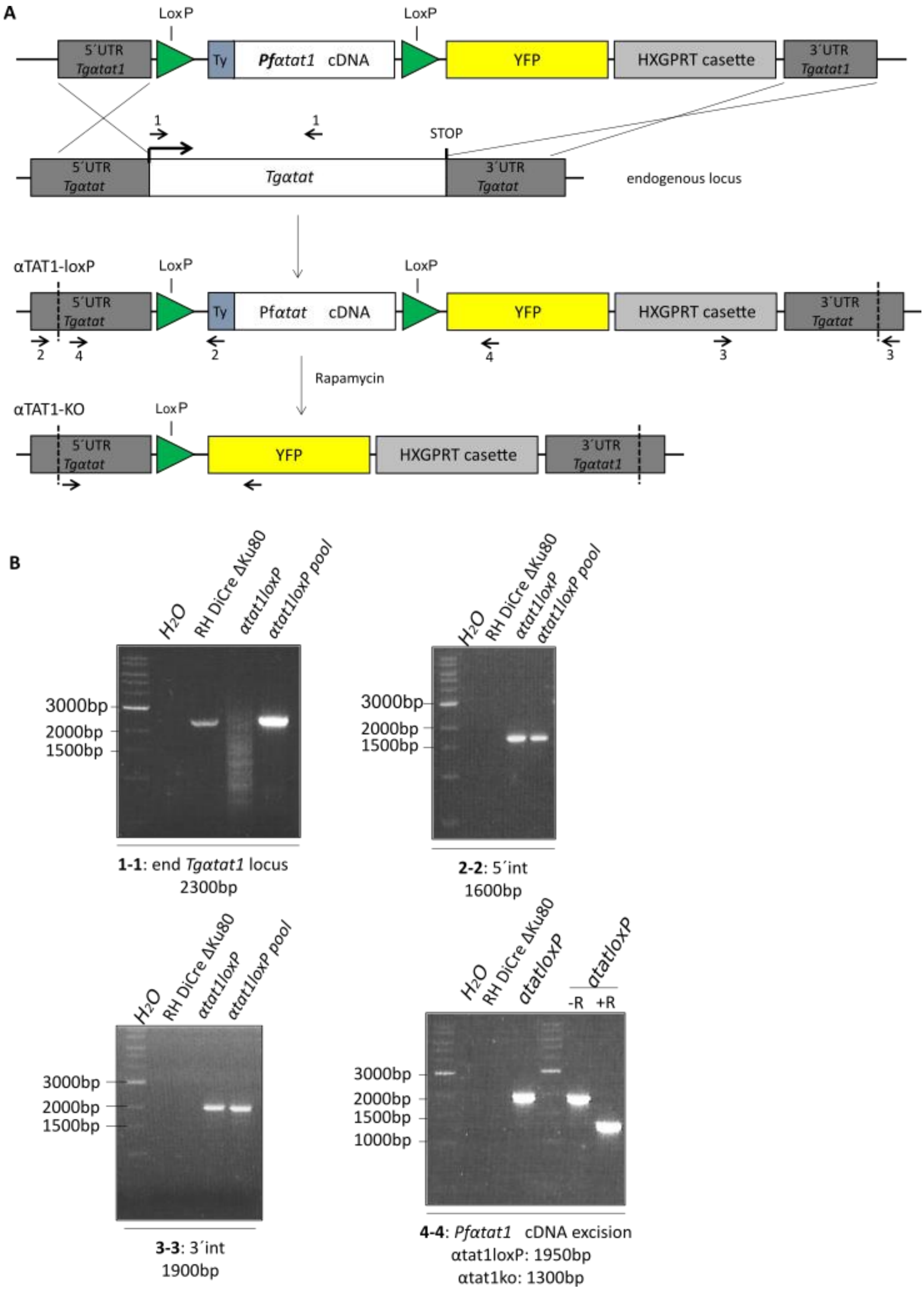

Supplementary figure 5: Generation of *T. gondii αTAT* loxP mutants. A) Schematic strategy depiction for generating *αtat1*loxP mutants. After linearization, the depicted strategy aimed at a site -specific integration of the Pf*αtat1*GeneSwap-construct into the endogenous Tg*mec17* locus of the parasite line RH DiCre ΔKu80 via double homologous recombination.

See text for details. Arrows represent primers applied to amplify the respective DNA fragment from parasitic genomic DNA. **B)** Genetic verification of the clonal *αtat1*loxP parasite line. Site specific integration of the Pf*αtat1*GeneSwap construct was verified applying different primer combinations. Depicted primer combinations and the respective DNA fragment size underneath the AGE pictures refer to the same primers shown in A. Primer combination 1-1 was applied to confirm the lack of the endogenous Tg*αtat1* locus in *αtat1*loxP parasites (far left panel). Primers 2-2 and 3-3 show site specific integration at the 5´and 3´UTRs, respectively (two middle panels). Finally, excision of Pf*αtat1* cDNA upon addition of rapamycin in *mec17*ko parasites was verified by primers 4-4 at 72 hours post induction (far right panel). RH DiCre ΔKu80: parental strain, *αtat1l*oxP: generated clonal *αtat1*loxP line; *αtat1*loxP Pool: parasite population obtained after transfection. H_2O: water control for PCR. Ladder: 1kb (New England Biolabs)

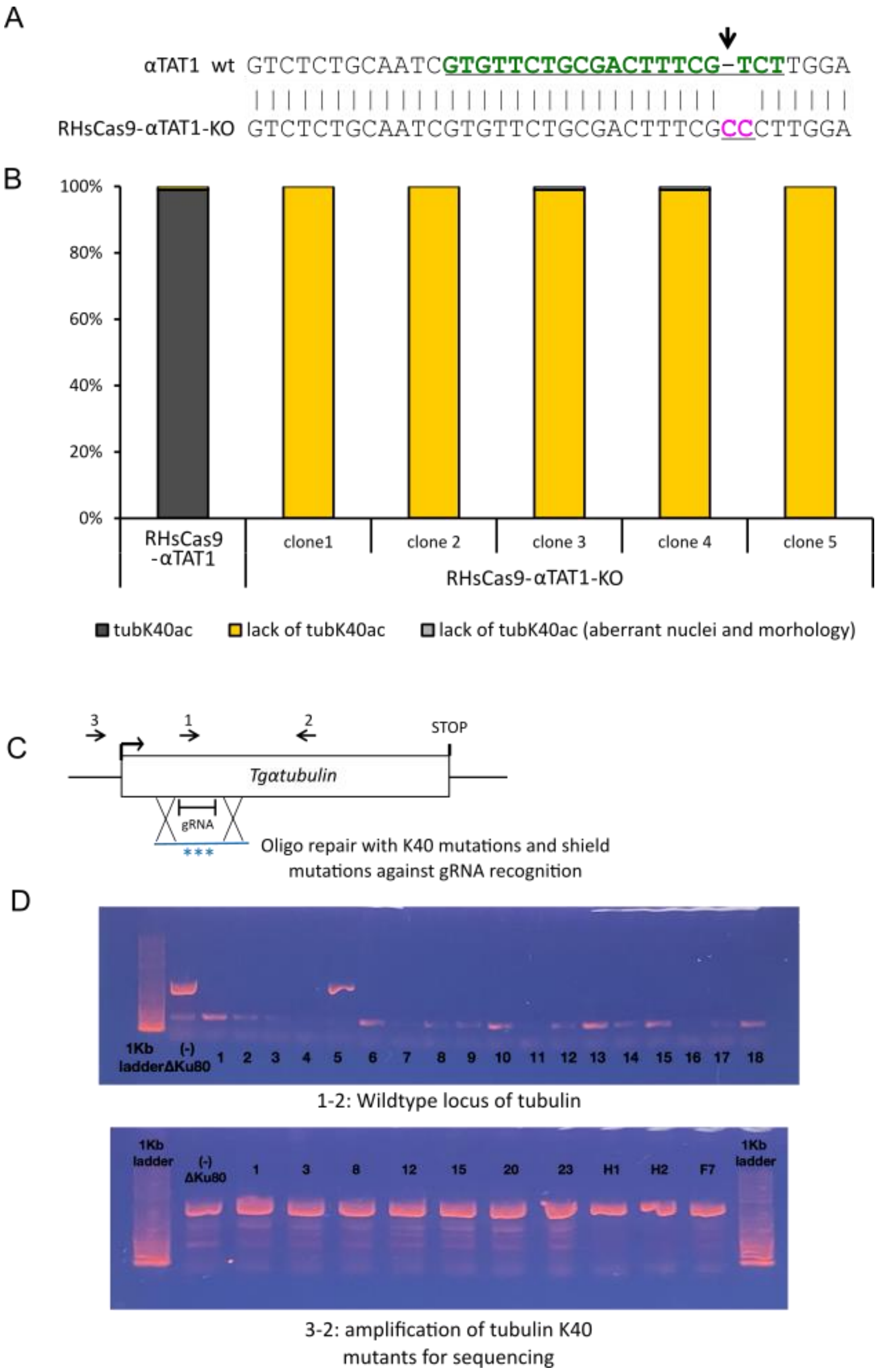

Supplementary figure 6: Generation and validation of *T. gondii* αTAT knockout and K40 mutant lines. A) Genome sequencing performed for the RHsCas9- αTAT -KO line (clone 1). Green and underlined letters indicate sgRNA sequence. Magenta and underlined letters represent nucleotide insertion in the mutant strain, causing a frame shift and, thus, the functional knock-out of the mec17 gene. The black arrow indicates the predicted cut side. **B)** Quantification of parasite vacuoles presenting aberrant nuclei and morphology in clonal

RHsCas9- αTAT-KO populations. Parasites were grown for 48h and fixed with 4 % PFA. IFA analysis was performed to allow for quantification. For each clone, one experiment was conducted (n=100). **C)** Schematic description of primers used to verify K40 mutants. **D)** Example of selection of K40 clones. Left: Agarose gel images showing the selection of clones lacking amplification of 1-2 probably due to insertion of shield mutations. Right: amplification of an α tubulin fragment containing the mutated region.

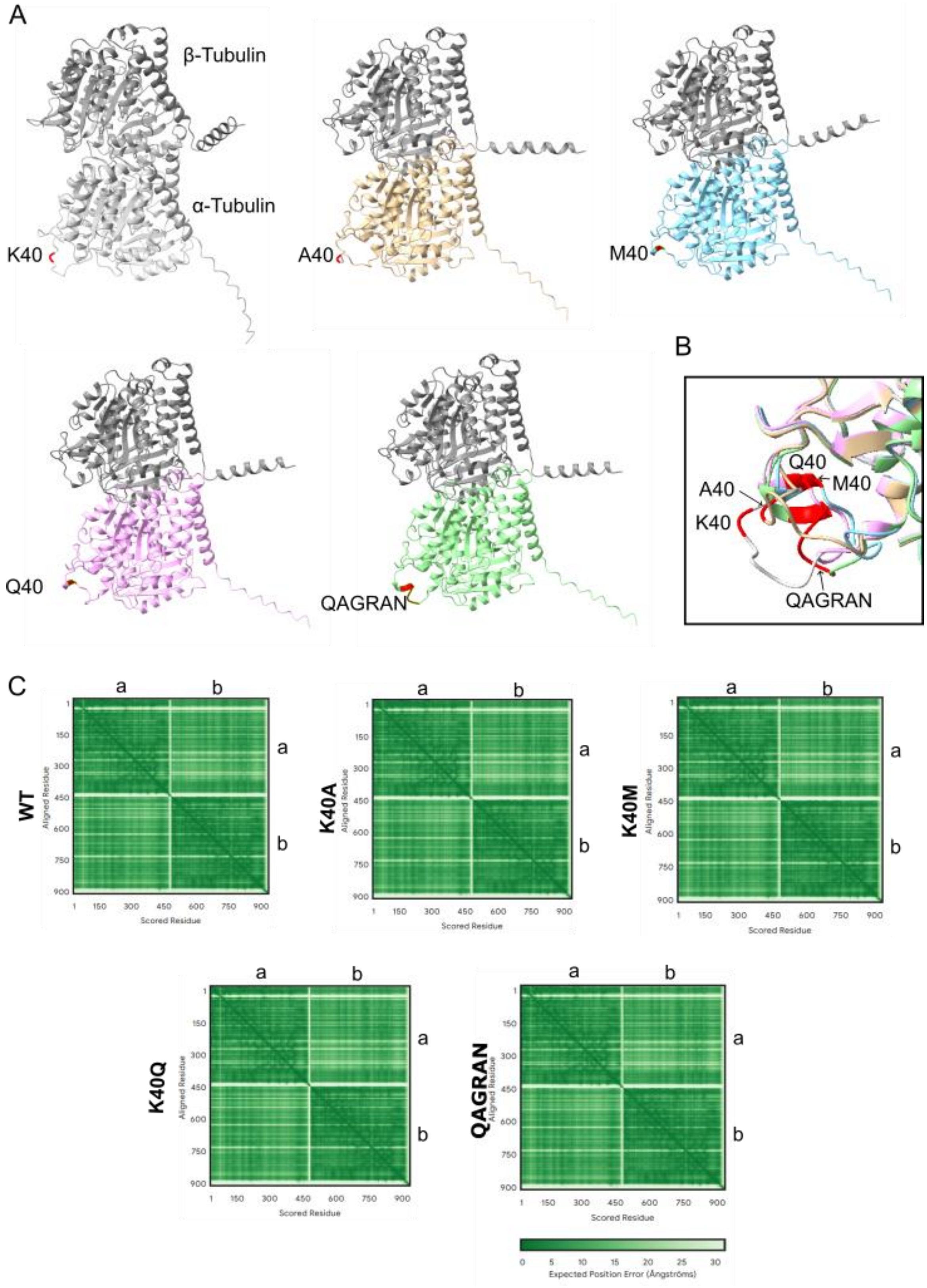

Supplementary Figure 7: Protein structure predictions of K40 mutated α1 and β tubulin in *T. gondii*. A) Protein structure prediction of the *T. gondii* WT tubulin dimer (gray) consisting of the β tubulin and the α tubulin. Amino acid position 40 with its lysine (K) residue or mutated residues are indicated in red. Mutated version K40A (yellow), K40M (blue), K40Q (magenta) and QAGRAN mutation (green). **B)** Superposition of mutated residues show change of structure in this region. **C)** Alignment and PAE score of all protein structures shown in A.
